# Macrophage Polarization and Alveolar Bone Healing Outcome: Despite a Significant M2 Polarizing Effect, VIP and PACAP Treatments Present a Minor Impact in Alveolar Bone Healing in Homeostatic Conditions

**DOI:** 10.3389/fimmu.2021.782566

**Published:** 2021-12-21

**Authors:** Michelle de Campos Soriani Azevedo, Angélica Cristina Fonseca, Priscila Maria Colavite, Jéssica Lima Melchiades, André Petenuci Tabanez, Ana Campos Codo, Alexandra Ivo de Medeiros, Ana Paula Favaro Trombone, Gustavo Pompermaier Garlet

**Affiliations:** ^1^ Bauru School of Dentistry, Department of Biological Sciences, University of São Paulo, Bauru, Brazil; ^2^ School of Pharmaceutical Sciences, Department of Immunology, São Paulo State University, Araraquara, Brazil; ^3^ Department of Health Sciences, Centro Universitário Sagrado Coração (UNISAGRADO), Bauru, Brazil

**Keywords:** VIP, PACAP, bone healing, M2 macrophages, osteoimmunology

## Abstract

Host inflammatory immune response comprises an essential element of the bone healing process, where M2 polarization allegedly contributes to a favorable healing outcome. In this context, immunoregulatory molecules that modulate host response, including macrophage polarization, are considered potential targets for improving bone healing. This study aims to evaluate the role of the immunoregulatory molecules VIP (Vasoactive intestinal peptide) and PACAP (Pituitary adenylate cyclase activating polypeptide), which was previously described to favor the development of the M2 phenotype, in the process of alveolar bone healing in C57Bl/6 (WT) mice. Experimental groups were submitted to tooth extraction and maintained under control conditions or treated with VIP or PACAP were evaluated by microtomographic (µCT), histomorphometric, immunohistochemical, and molecular analysis at 0, 3, 7, and 14 days to quantify tissue healing and host response indicators at the healing site. Gene expression analysis demonstrates the effectiveness of VIP or PACAP in modulating host response, evidenced by the early dominance of an M2-type response, which was paralleled by a significant increase in M2 (CD206^+^) in treated groups. However, despite the marked effect of M1/M2 balance in the healing sites, the histomorphometric analysis does not reveal an equivalent/corresponding modulation of the healing process. µCT reveals a slight increase in bone matrix volume and the trabecular thickness number in the PACAP group, while histomorphometric analyzes reveal a slight increase in the VIP group, both at a 14-d time-point; despite the increased expression of osteogenic factors, osteoblastic differentiation, activity, and maturation markers in both VIP and PACAP groups. Interestingly, a lower number of VIP and PACAP immunolabeled cells were observed in the treated groups, suggesting a reduction in endogenous production. In conclusion, while both VIP and PACAP treatments presented a significant immunomodulatory effect with potential for increased healing, no major changes were observed in bone healing outcome, suggesting that the signals required for bone healing under homeostatic conditions are already optimal, and additional signals do not improve an already optimal process. Further studies are required to elucidate the role of macrophage polarization in the bone healing process.

## Introduction

Bone is a dynamic tissue that provides mechanical support and protection to organs and tissues, and serves as a mineral reservoir to body physiological activities. Bone properties are closely related to its high remodeling capacity, which allows its adaptation to growth and mechanical stimuli, the regulation of mineral components storage/mobilization depending on physiological demand, and confers to the bone tissue a significant healing capacity (1–3). The interplay between osteoblasts, osteocytes, and osteoclasts is required to maintain bone homeostasis and function, and also to restore bone integrity in response to injury. Importantly, bone cells are susceptible to the influence of numerous endocrine and immune system derived factors, which can influence bone homeostasis and healing capacity (1–3).

Regarding the interaction between bone and immune systems, bone healing allegedly depends on a transitory inflammatory process, where innate immune system cells migrate to the injury site to remove cell or matrix debris and to regulate tissue healing *via *the production of inflammatory mediators and growth factors (4). Although the exact contribution of leukocytes subsets to the bone healing process remains unknown, studies point to macrophages as key elements of general tissue healing (5). While classically considered as pro-inflammatory cells, macrophages may present distinct functional phenotypes, termed M1 and M2 (6, 7). The original definition of M1 and M2 macrophage derives from the effects of prototypic Th cytokines (8, 9); the M1 differentiation is driven by IFN-γ and results in a pro-inflammatory phenotype; while M2 regulatory and pro-reparative phenotype is induced by IL-4 (6, 7, 10–12).

Interestingly, general tissue healing seems to involve an initial M1 polarization that typically shifts toward the pro-reparative M2 phenotype over time (13, 14). The initial M1 phenotype is supposed to contribute to healing releasing pro-inflammatory cytokines (such as TNF-α and IL-1β), triggering leukocytes chemoattraction to the injury site, while the subsequent contribution of M2 macrophages theoretically involve the production of angiogenic and growth factors required to tissue healing (8, 15, 16). At this point, it is mandatory to mention that the exact contribution of macrophage subsets to healing outcomes is still elusive. Studies demonstrate that the unremitting pro-inflammatory response, associated with the M1 phenotype, is usually associated with chronic wounds and impaired healing, suggesting that exacerbated M1 responses may present a detrimental role in healing outcomes (13, 17). In this context, the shift of the injury site microenvironment towards an M2 profile is essential for a proper healing outcome and would comprise a potential therapeutic strategy to promote and/or improve the healing process (18–21).

Similar to the general tissue healing, the immune response at bone injury sites seems to involve initially the predominance of pro-inflammatory cytokines associated with the presence of M1 macrophages, followed by the subsequent production of anti-inflammatory factors and the transition to an M2 profile dominance (13). Indeed, M1 to M2 transition is regarded as an important event of bone healing, associated with the inflammation resolution at healing sites, being a persistent or non-resolving inflammatory response associated with impaired healing (17). However, few studies have focused on the potential role of macrophage subsets in bone healing, and the majority of data in the field is essentially associative (22–24). Therefore, cause-and-effect studies are required to determine the real impact of M1/M2 balance in bone healing outcomes. It is also mandatory to consider that since macrophage polarization occurs in the response site, the local microenvironment theoretically has a determining role in the polarization outcome. However, the prototypic Th cytokines (i.e., IFN-γ and IL-4) originally described as macrophage polarization inducers (7, 10, 12), are usually absent or present in very low levels in bone healing microenvironment, possibly due to the relative absence of T cells in such sites (25); consequently, the factors responsible for the determination of M1/M2 dynamics along bone healing remain unclear.

In this context, recent studies describe other molecules capable of modulating macrophages polarization, such as the VIP–PACAP system. VIP (vasoactive intestinal peptide) and PACAP (pituitary adenylate cyclase activating polypeptide) are described as highly homologous peptides (68% homology), which once released by local innervation or immune cells, present immunoregulatory properties over multiple cell types, namely, macrophages and bone cells (26–29). The VIP–PACAP system comprises the receptors VPAC1 and VPAC2, both presenting high affinity for VIP and PACAP; and PAC1, an exclusive PACAP receptor (30).

VIP presents a broad spectrum of biological functions that include the modulation of both innate and adaptive immunity, due to the inhibitory actions in signaling cascades involved in the production of several pro-inflammatory mediators (31) and the negative regulation of co-stimulatory signals from macrophages (28, 32–34). Similar to VIP, PACAPs (30) also are capable of influencing immunological system elements (32, 33), being previously described as a macrophages deactivator (28, 35–37), leading to the inhibition of pro-inflammatory cytokines and the stimulation of anti-inflammatory cytokines (31). Importantly, since such studies were performed prior to the M1/M2 description, the deactivating features are compatible with an M1 to M2 macrophages conversion (38). In addition to the immune system related function, VIP and PACAP present a protective effect in bone associated inflammation (35, 36), reinforcing its potential involvement in the modulation of inflammation–bone healing interplay (36, 37, 39–41).

Therefore, the aim of this study was to take advantage of the immunomodulatory properties of VIP and PACAP, which have been sown to favor the development of the M2 phenotype (32, 42–44), to investigate the impact of M2 response in bone healing outcome in mice, and also investigate the potential mechanisms/pathways underlying VIP and PACAP immunoregulatory effects in bone healing sites.

## Materials and Methods

### Animals

The experimental groups consisted of 8-week-old wild type (WT) mice acquired from the Ribeirão Preto Medical School (FMR/USP) breeding facility, maintained during the experimental period in the facility of the Department of Biological Sciences of FOB/USP. During the study period, the mice were fed with a standard sterile solid feed of mice (Nuvital, Curitiba, PR, Brazil) and sterile water. The experimental protocol was approved by the Institutional Committee for Care and Animal Use and by Guide for the Care and Use of Laboratory Animals (CEEPA-FOB/USP, process # 001/2016).

### Experimental Protocol and Mice Tooth Extraction Model

Male and female (on the same proportion) C57BL/6 wild type (WT) mice, were treated (experimental groups) or not (control group) with VIP (Sigma Aldrich—Catalog number V6130—0.05 mg/kg IP, 24/24 h) or PACAP (Bachem—Catalog number H-8430—0.05 mg/kg IP, 24/24 h), beginning 1 day prior to the upper right incisor extraction and throughout the experimental periods (0, 3, 7 and 14 days post tooth extraction), subsequently, were anesthetized by intramuscular administration of 80 mg/kg of Ketamine Chloride (Dopalen, Agribrans Brasil Ltda) and 160 mg/kg of Xylazine Chloride (Anasedan, Agribrands Brasil Ltda) in the proportion 1:1 according to the animal body mass. At the end of the experimental periods, animals were euthanized with an excessive dose of anesthetic, and the maxillae were collected (N = 5 group/time) for microtomographic (μCT) samples, and then prepared for histomorphometry, immunohistochemical, and collagen birefringence analysis or submitted to molecular analysis by Real Time PCR Array (N = 4 group/time).

### Micro-Computed Tomography (μCT) Assessment

The samples were scanned by the Skyscan 1174 System (Skyscan, Kontich, Belgium), at 50 kV, 800 μA, with a 0.5 mm aluminum filter and 15% beam hardening correction, ring artifacts reduction, 180° of rotation and exposure range of 1°. Images were captured with 1,304 × 1,024 pixels and a resolution of 14 μm pixel size. Projection images were reconstructed using the NRecon software and three-dimensional images obtained by the CT-Vox software. Morphological parameters of trabecular bone microarchitecture were assessed using the CTAn software in accordance with the recommended guidelines (45). A cylindrical region of interest (ROI) with an axis length of 3 mm and a diameter of 1 mm was determined by segmenting the trabecular bone located from the coronal to apical thirds. Measurements included the tissue volume (TV), bone volume (BV), bone volume fraction (BV/TV, %), trabecular thickness (Tb.Th, mm), trabecular number (Tb.N, mm) and trabecular separation (Tb.Sp) (25, 45–47).

### Histomorphometric Analysis

Serial sections (8 semi-serial sections of each maxilla, with a 5 μm thickness for each section) were obtained using a microtome (Leica RM2255, Germany) and stained with HE (hematoxylin and eosin). Morphometric measurements were performed by a single calibrated investigator with a binocular light microscope (Olympus Optical Co, Tokyo, Japan) using a 100× immersion objective and a Zeiss kpl 8× eyepiece containing a Zeiss II integration grid (Carl Zeiss Jena GmbH, Jena, Germany) with 10 parallel lines and 100 points in a quadrangular area. The grid image was successively superimposed on approximately 13 histological fields per histological section, comprised of all tooth sockets from the coronal limit adjacent to the gingival epithelium until the lower apical limit. For each animal/socket, sections from the medial were evaluated. In the morphometric analysis, points were counted coinciding with the images of the following components of the alveolar socket: clot, inflammatory cells, blood vessels, fibroblasts, collagen fibers, bone matrix, osteoblasts, osteoclasts and other components (empty space left by extracellular liquid and bone marrow); similar to previous descriptions (25, 47–50). The results were presented as the mean of volume density for each evaluated structure.

### Collagen Birefringence Analysis

The Picrosirius-polarization stain method and the quantification of birefringent fibers were performed to assess the structural changes in the newly formed bone trabeculae matrix, based on the birefringence of the collagen fiber bundles as previously described (25, 47, 51, 52). Serial sections (8 semi-serial sections of each maxilla) with 5 μm thickness were cut and stained by Picrosirius Red; all sections were stained simultaneously to avoid variations due to possible differences in the staining process. Picrosirius Red-stained sections were analyzed through a polarizing lens coupled to a binocular inverted microscope (Leica DM IRB/E), and all images were captured with the same parameters (the same light intensity and angle of the polarizing lens 90° to the light source). AdobePhotoshopCS6 software was used to delimit the region of interest (an alveolar area comprised of new tissue with the external limit comprised of the alveolar wall). The quantification of the intensity of birefringence brightness was performed using the AxioVision 4.8 software (Carl Zeiss, Oberkochen, Germany). For quantification, the images were binarized for definition of the green, yellow and red color spectra, and the quantity of each color pixels^2^ corresponding to the total area enclosed in the alveoli was measured. Mean values of 4 sections from each animal were calculated in pixels^2^.

### Immunohistochemistry Analysis

Histological sections were deparaffinized following standard procedures. For antigen retrieval, citrate buffer solution was used in a steamer (96/98°C, 30’). After that, the material was incubated with 3% Hydrogen Peroxidase Block (Spring Bioscience Corporation, CA, USA) and subsequently with 7% NFDM to block serum proteins. The histological sections from all of the groups were incubated with Ly6g-Gr1 polyclonal antibody—sc-168490 (Santa Cruz Biotechnology, Santa Cruz, CA, USA), F4/80 (A-19) polyclonal antibodies—sc-26642 (Santa Cruz Biotechnology, Santa Cruz, CA, USA), B7-1 CD80 (H-208) monoclonal antibody—sc-9091 (Santa Cruz Biotechnology, Santa Cruz, CA, USA), CD206 (C-20) polyclonal antibody—sc-34577 (Santa Cruz Biotechnology, Santa Cruz, CA, USA), VIP (M19) polyclonal antibody—sc-7841 (Santa Cruz Biotechnology, Santa Cruz, CA, USA), PACAP (C-19) polyclonal antibody—sc-7840 (Santa Cruz Biotechnology, Santa Cruz, CA, USA), CD3-E (M-20) polyclonal antibody—sc-1127 (Santa Cruz Biotechnology, Santa Cruz, CA, USA) at manufacturer’s indication concentrations for 1 h at room temperature. The identification of antigen–antibody reaction was performed using 3-3’-diaminobenzidine (DAB) and counter-staining with Mayer’s hematoxylin. Positive controls were assessed in the mouse spleen for positive Gr1, F4/80, CD80, CD206, CD3, PACAP, and VIP. Negative controls were also assessed in mouse spleen samples representing the 3 groups studied, which submitted to all procedures, with the exception of primary antibodies in order to rule out any possible cross-reactions. The analysis of immunolabeled cells was performed by a single calibrated investigator with a binocular light microscope (Olympus Optical Co., Tokyo, Japan) using a 100× immersion objective. The quantitative analysis for the different markers was performed throughout the alveolar extension. The absolute number of immunolabeled cells was obtained to calculate the mean for each section.

### Real Time PCR Array Reactions

Real Time PCR array reactions were performed as previously described (25, 47). In short, extraction of total RNA from the remaining alveolus was performed with the RNeasy Plus kit (Qiagen Inc, Valencia, CA) according to the manufacturer’s instructions. The integrity of the RNA samples was verified by analyzing 1 µl of the total RNA in a 2100 Bioanalyzer (Agilent Technologies, Santa Clara, CA) according to the manufacturer’s instructions, and the complementary DNA (cDNA) was synthesized using 1 mg of RNA through a reverse transcription reaction QuantiTect Rev Transcription kit (Qiagen Inc, Valencia, CA). Real Time PCR array was performed in a Viia7 instrument (Thermo Fisher Scientific, Carlsbad, CA) using a custom panel containing targets “Wound Healing” (PAMM-121), “Inflammatory cytokines and receptors” (PAMM-011), and “Osteogenesis” (PAMM-026) (SA Biosciences, Frederick, MD) for gene expression profiling. Real Time PCR array data were analyzed by the RT2 profiler PCR Array Data Analysis online software (SA Biosciences, Frederick, MD) for normalizing the initial geometric mean of three constitutive genes (GAPDH, ACTB, Hprt1) and subsequently normalized by the control group, and expressed as fold change relative to the control group.

### Statistical Analysis

Data were presented as means ± SD, initially, the data distribution were tested by the Kolmogorov–Smirnov normality test. The statistical significance inside the group was analyzed by a Kruskal–Wallis test followed by a Dunn’s posttest or by a Mann–Whitney test, while the statistical significance between periods was analyzed by Student’s t-tests. Real Time PCR array data were analyzed by a Mann–Whitney test followed by a Benjamini–Hochberg test. Values of p <0.05 were considered statistically significant. Both were performed with Graph-Pad Prism 7.0 software (GraphPad Software Inc, San Diego, CA).

## Results

### Molecular Analysis Using Real Time PCR Array

**Figure 1:**
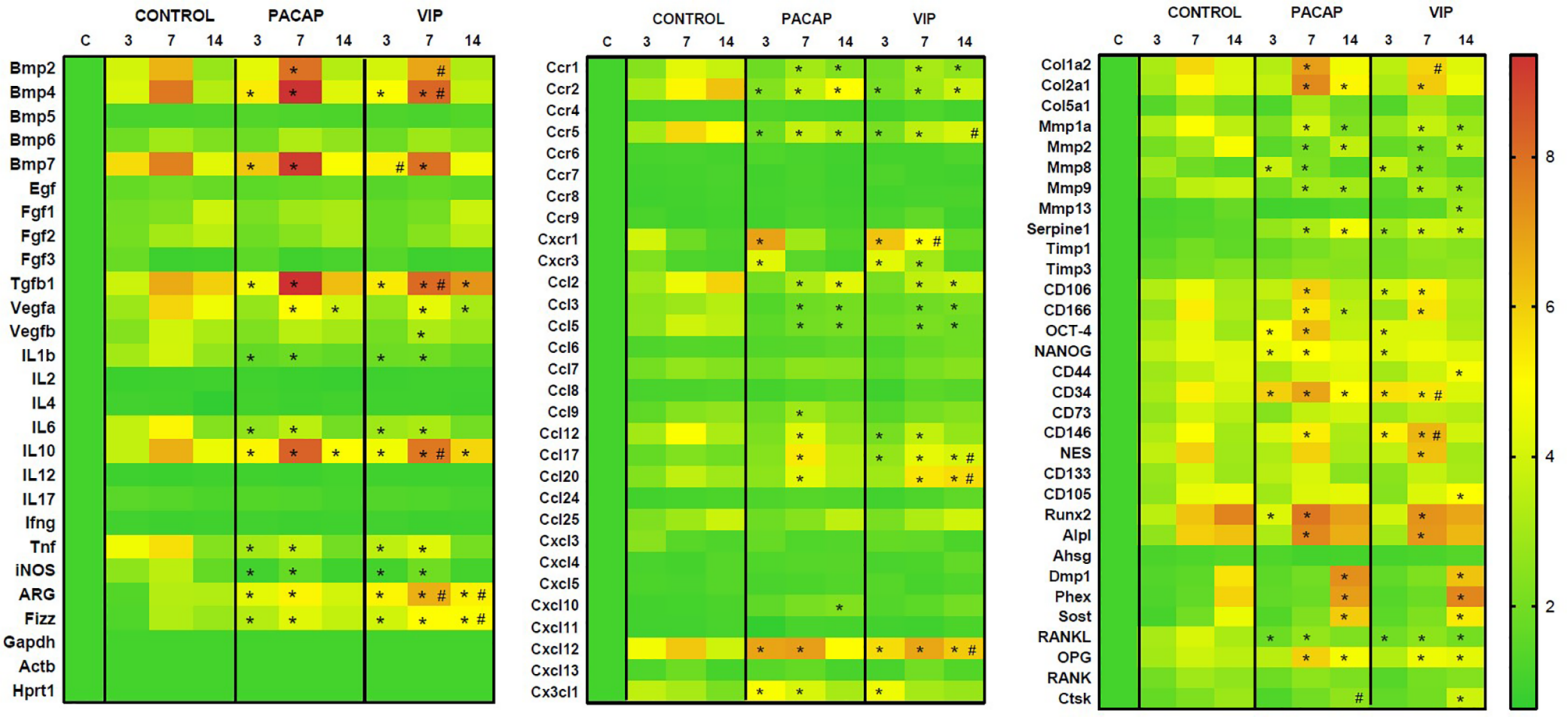
Molecular analysis (Real Time PCR Array) using heat map to quantify the expression of the growth factors, extracellular matrix markers, bone markers and cytokines in bone healing process among control, VIP and PACAP treated groups. Results were obtained when comparing the relative expression of the different groups norm normalized by Gapdh, Actb, and Hprt1 (* represents the differences between the control group; # represents the difference between the VIP and PACAP groups).

The gene expression of several molecules is involved in inflammatory response and bone healing, considering growth factors, immunological/inflammatory markers, extracellular matrix, and bone markers was investigated employing a pool of samples from 3, 7 and 14-day periods analyzed using Real Time PCR array (**Figure 1**). Among several growth factors, the VEGFA (7–14 days) expressions were downregulated in the VIP- and PACAP-treated groups in relation to the control group, and the same was observed in the VEGFB expression (7 days) only on VIP-treated group. In addition to these growth factors, BMP2 (7 days), BMP4 (3–7 days), and BMP7 (3–7 days) were upregulated in both treated groups when compared to the control group and superior in VIP compared to PACAP on BMP2 and BMP4 on 7 days and BMP7 on 3 days of treatment. Considering the immunological markers analyzed (cytokines, chemokines, chemokine receptors, and inflammatory mediators) the expression of IL-1b, IL-6, TNF, and iNOS were downregulated in the VIP and PACAP groups (3–7 days), while TGF-β1, IL-10, ARG, and Fizz were upregulated in the analyzed periods in the treated groups. In respect to CCR1, CCR2, CCR5, CCL2, CCL3, CCL5, and CCL12 these were downregulated in VIP and PACAP, and CXCR1,CXCR3,CCL9, CCL17, CCL20, CXCL12, and CX3CL1 were upregulated in the treated groups, and also CXCL10 in the VIP-treated group at 14 days. Subsequently, numerous extracellular matrix markers, including Col1a2, Col2a1, MMP8, SERPINE1, CD106, CD166, OCT-4, NANOG, CD34, CD146, NES, Runx2, Alpl, Dmp1, Phex, Sost, and OPG were found to be upregulated in VIP/PACAP-treated groups; while MMP1a, MMP2, MMP9 and RANKL were downregulated in the treated groups. Regarding MMP13, CD44, CD105, and CTSK these were upregulated in the VIP-treated group.

### Immunohistochemistry Analysis of Ly6g-Gr1, F4/80^+^, CD80-B7-1^+^, CD206^+^ and CD3^+^ Cells

**Figure 2:**
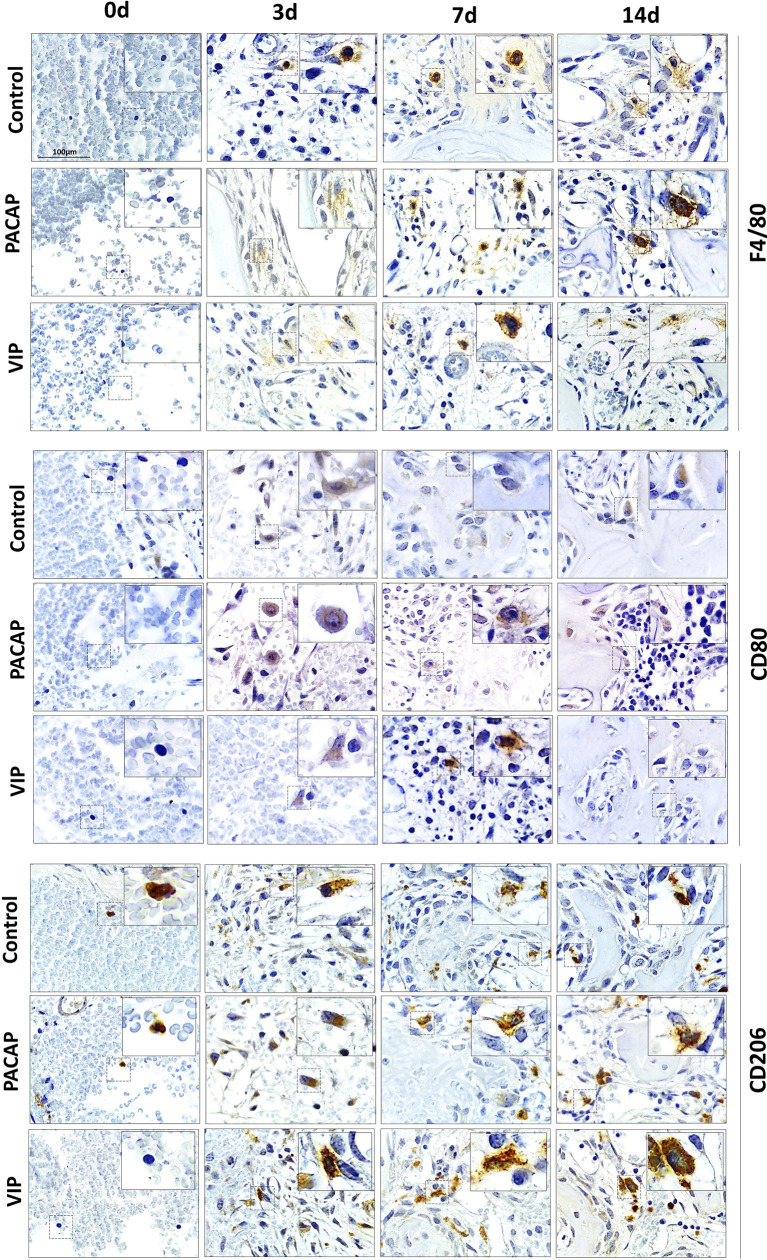
Immunohistochemistry analysis for F4/80^+^, CD80^+^, and CD206^+^ cells present in the bone repair process in control, VIP and PACAP treated groups. Representative sections from medial thirds of the socket at days 0, 3, 7 and 14 days after tooth extraction. Anti-staining Mayer’s hematoxylin; objective of 100×.

**Figure 3:**
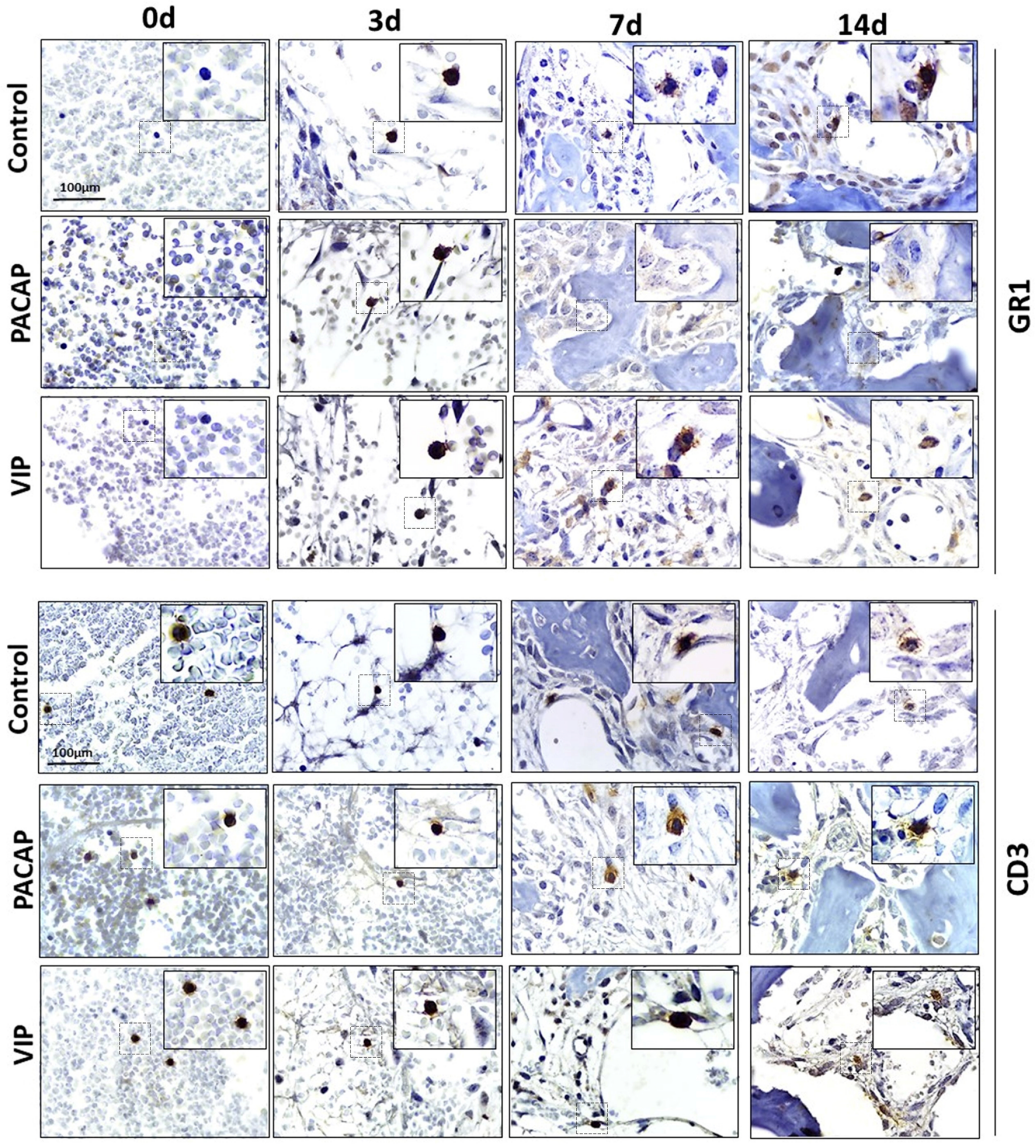
Immunohistochemistry analysis for Ly6g-GR1^+^ and CD3^+^ cells present in the bone repair process in control, VIP and PACAP treated groups. Representative sections from medial thirds of the socket at days 0, 3, 7 and 14 days after tooth extraction. Anti-staining Mayer’s hematoxylin; objective of 100×.

**Figure 4:**
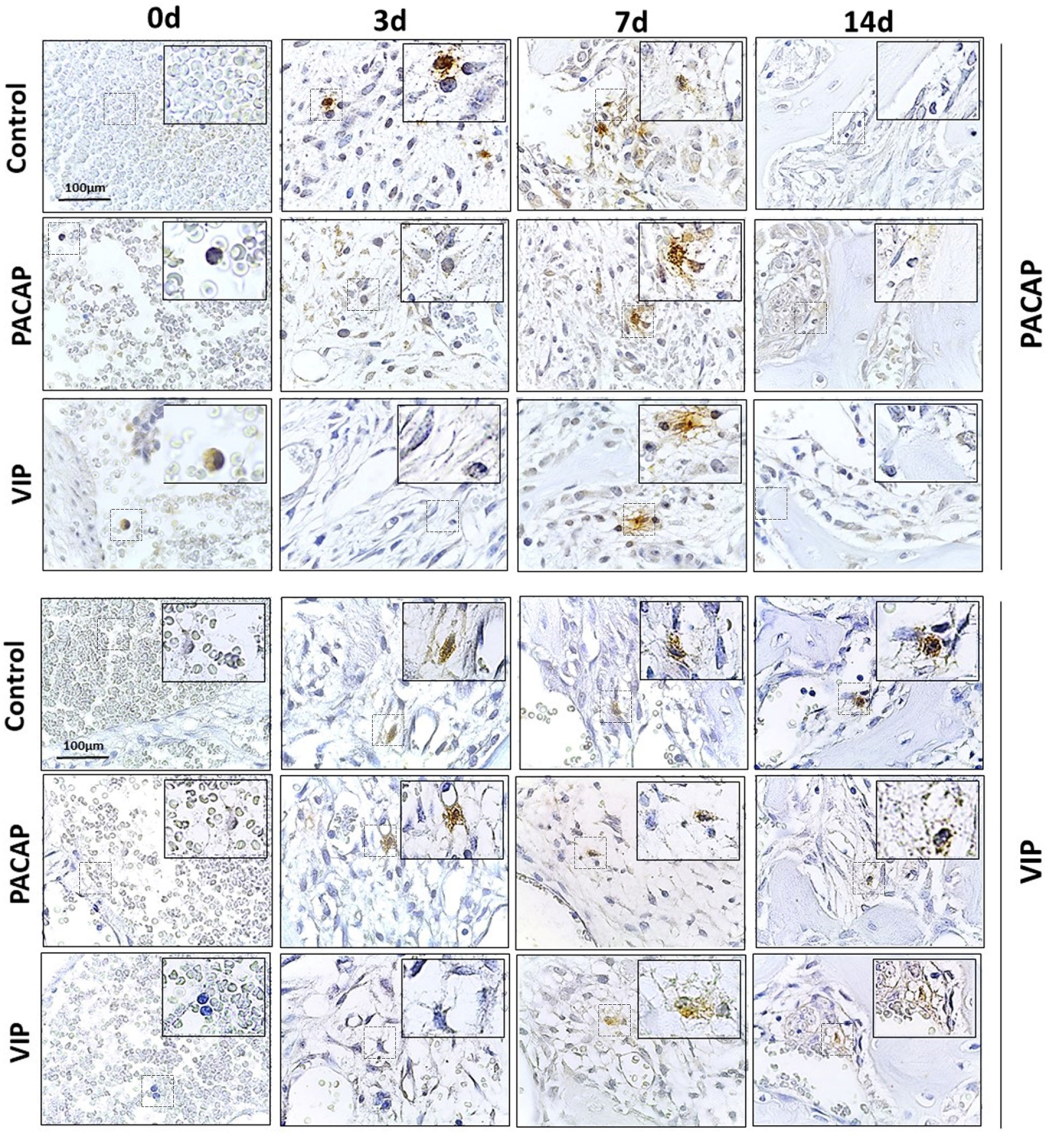
Immunohistochemistry analysis for VIP+ and PACAP+ cells present in the bone healing process in the control, VIP and PACAP treated mice. Representative sections from medial thirds of the socket at days 0h, 3, 7 and 14 days after tooth extraction. Anti-staining Mayer hematoxylin; objective of 100×.

**Figure 5:**
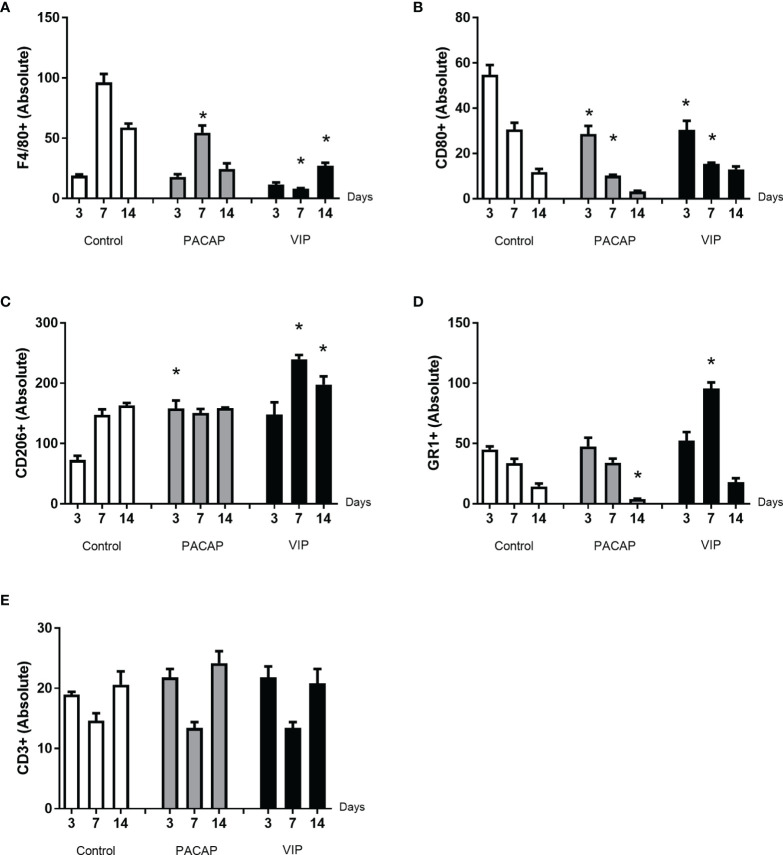
Analysis of inflammatory cells in the alveolar bone healing kinetics after tooth extraction in control, VIP and PACAP treated mice. Immunohistochemistry quantification corresponding **(A)** F4/80^+^, **(B)** CD80^+^, **(C)** CD206^+^, **(D)** Ly6g-Gr1^+^, and **(E)** CD3^+^ (* represents the differences between the control group).

To verify the influence of macrophages and other inflammatory cells in the healing site, the immunolabeling was performed to F4/80, CD80 (B7-1), CD206 (**Figure 2**), CD3, and GR1(Ly6g) targets (**Figure 3**). In addition, VIP and PACAP markers (**Figure 4**) were included to verify the cell production of these neuropeptides during the healing process, and also to verify the efficiency of the antagonist in the receptors VPAC1 and VPAC2. Each marker was analyzed separately to confirm how a marker for undifferentiated macrophages (M0) used the F4/80 target. For this marker, the number of immunostained cells was, in general, superior in the control group in the 7-day period for the PACAP group and 7 and 14 days for the VIP-treated group when compared with the control group, presenting a significantly higher number of stained cells (**Figure 5A**).

Considering CD80^+^ cells (M1 macrophages), a decreasing number of immunolabeled cells after 3 days in the control group were observed which cannot be observed in the treated groups. In addition, a small number of cells were observed in all periods in relation to the control with significant differences and 3 and 7 days to both treated groups. Also, the number of cells stained in the group that received the treatments with VIP and PACAP was higher (**Figure 5B**).

For CD206^+^ immunolabeled cells (M2 macrophages), a progressive increase was observed in the control group. In the VIP group, the number of immunostained cells was higher compared to the control with the highest number verified in the VIP treated group and there was a significant increase in 7 and 14 days in comparison with the control group. On the other hand, a decrease occurred in 14 days in the treated groups unlike that observed in the control group. A similar increase happened in the PACAP treated group in 3 days when compared with the control group; however, the number of cells remained similar in the subsequent 7- and 14-day periods (**Figure 5C**).

For the GR1 target, in the control group, the highest number of cells was observed in 3 days which gradually decreased in 7 and 14 days. For the group treated with VIP, the number of positive cells was significantly higher at 7 days when compared with the control. In the group treated with PACAP, the number of positive cells was higher in 3 and 7 days, when compared with the control group and statistically decreased in 14 days when correlated with the control group (**Figure 5D**).

When CD3-labeled cells were analyzed, the number of labeled cells in the control group was similar in all periods. In the group treated with VIP, the kinetics was similar to the control group, but marked cells were more observed at 3 and 14 days in relation to the control group with no significant differences between groups. In the group treated with PACAP, no differences were attended (**Figure 5E**).

### Immunohistochemistry Analysis of PACAP^+^ and VIP^+^ Cells

**Figure 6:**
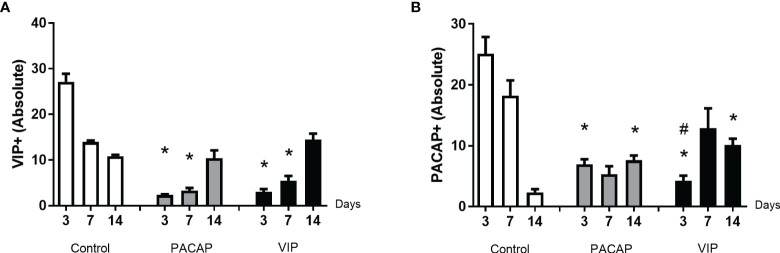
Analysis of inflammatory cells in the alveolar bone healing kinetics after tooth extraction in control, VIP and PACAP treated mice. Immunohistochemistry quantification corresponding **(A)** VIP^+^ and **(B)** PACAP^+^ (* represents the differences between the control group; # represents the difference between VIP and PACAP group).

In the cells labeled for VIP, the expression of positive cells was higher in the control group compared to the VIP group, with a significantly higher number of cells immunolabeled in 3 and 7 days (**Figure 6A**). In the cells labeled for PACAP, the number of positive cells in the control group was significantly higher in the period of 3 days when compared to the treated groups, and significantly decreased in the 14 days compared to the VIP-treated group (**Figure 6B**). Due to their homology with PACAP, cells labeled for VIP were also evaluated in the PACAP-treated group. The expression of positive cells increased in the control group, with a higher number of cells immunolabeled in 3 days. In the treated group the expression was lower in the 3- and 7-day period when compared with the control group, except in the 14 days. In relation to PACAP positive cells, in the control group, the highest number was observed in the 3-day period, followed by a decrease in 7 and 14 days. In its turn, a decreased number of positive cells in 3 and 7 days were shown in the PACAP treated group, followed by a significant increase in 14 days, when compared with the control group (**Figure 6B**).

### Comparative Histomorphometric Analysis

#### Connective Tissue, Clot, and Other Structures

**Figure 7:**
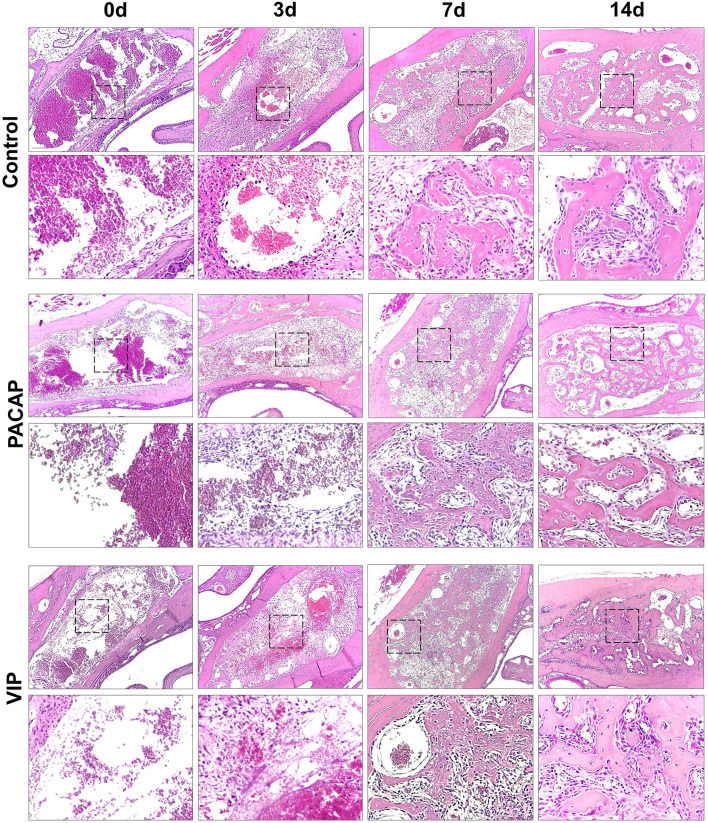
Histological analysis of the alveolar healing process in control mice or treated with PACAP or VIP, in the 0h, 3-, 7-, and 14-day post-exodontia periods. Photomicrographs representing the average region of the dental alveolus in 10× magnification; histological sections obtained after 0h, 3, 7 and 14 days after exodontia; HE stain.

**Figure 8:**
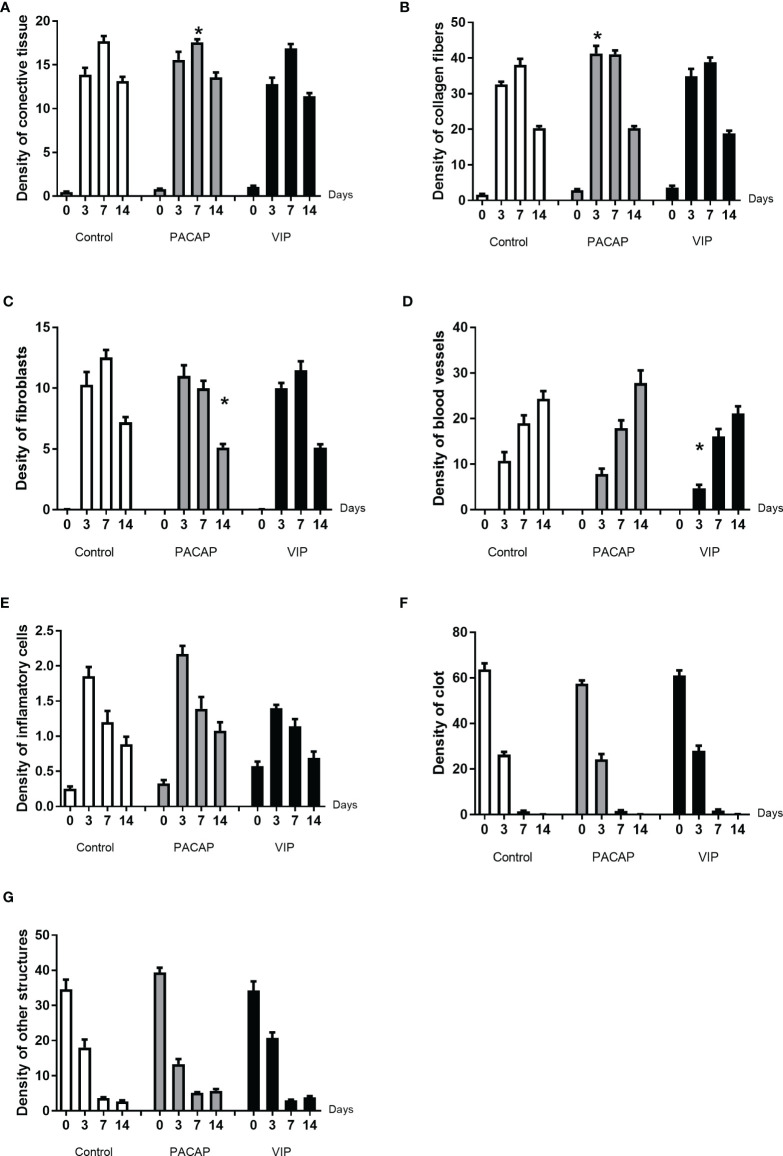
Comparative density analysis (%) of connective tissue **(A)** and its components **(B–G)** present in the dental alveolus of control mice and treated with VIP and PACAP in the 0, 3-, 7-, and 14-day post-exodontia periods. Results are presented as the means of density for each structure of the alveolar socket: collagen fibers, fibroblasts, blood vessels, inflammatory cells, clot and other components (empty space left by intercellular liquid and spaces). Also, the results depict the total density of connective tissue (represented by the sum of collagen fibers, fibroblasts, blood vessels, and inflammatory cells). The results represent the values of the mean and standard deviation in each of the periods analyzed (* represents the differences between the control group).

Based on the histomorphometric analysis, our results show a similar pattern in the healing process between the control and the treated groups (**Figure 7**). Regarding the kinetics of the healing, the control and the treated groups showed a progressive increase of the connective tissue starting in the 3rd to 7th days followed by a small decrease in the 14th day in the analyzed groups. Additionally, when the total area of connective tissue was measured, no significate differences were observed between the control and the treated groups (**Figure 8A**). Considering the density of collagen fibers, similar to the previous parameter, there was a progressive increase, starting in the 3rd day followed by a decrease on the 14th day; compared to the control group, a significant difference was observed in the PACAP group with an increase in the 3-day period (**Figure 8B**).

Regarding the density of fibroblasts occupied area (**Figure 8C**), in the control and the treated groups, an increase was observed at 3 to 7 days, followed by a decrease in 14 days, with no significant differences between VIP-treated and control groups. On the other hand, in the group treated with PACAP the density of fibroblasts was higher in 3 days with a decrease in the 7 to 14-day period. In addition, the PACAP group showed observable significant differences in relation to the control group in the 14-day period. Regarding the blood vessels area density, similar to parameters previously mentioned about the kinetics, the area occupied by blood vessels increased gradually from 3 to 14 days, with a significant difference between the VIP treated group and the control group in the 3-day experimental period, being smaller in the VIP-treated group. There was no difference between the PACAP group (**Figure 8D**).

As regards the area of inflammatory infiltrate, when the control and the VIP groups were compared although, with fewer cells, similar kinetics could be observed. It is of note that there was an increase in all the groups between the periods 0 to 3 days. After this initial process, in the 14-day period, as was expected, a decrease in the density of inflammatory cells with no differences between the control and the treated groups was observed (**Figure 8E**). Analyzing the blood clot density, it was observed that there was no difference between the control and the groups that received treatment presenting similar kinetics in all groups, starting with a peak of a blood clot in 0-day followed by an intense decrease (**Figure 8F**). In relation to the parameter called “other structures”, differences were not observed between the groups. This parameter refers to the empty spaces occupied by interstitial fluid, which gradually decreased over the periods in all groups evaluated (**Figure 8G**)

#### Bone Tissue Structures

**Figure 9:**
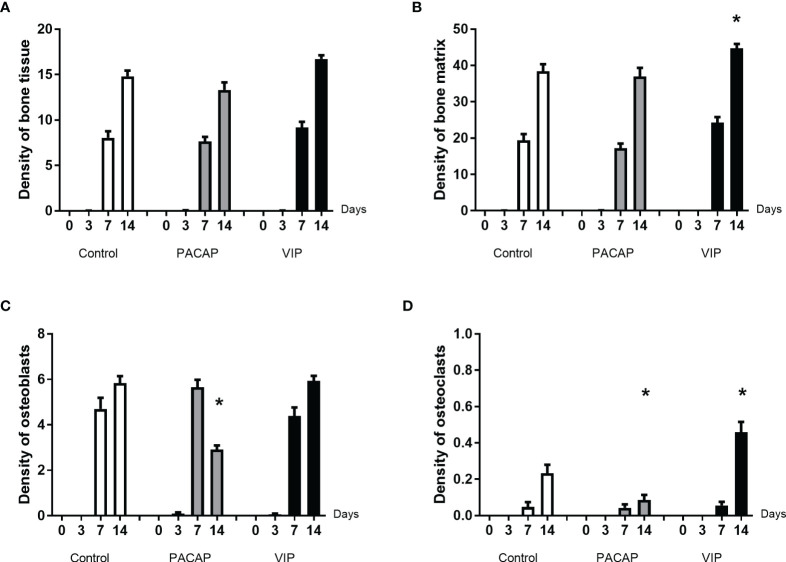
Comparative analysis of area density (%) of bone tissue **(A)** and its components **(B–D)** present in the dental alveoli of control mice and treated with VIP and PACAP in the 0, 3-, 7-, and 14-day post-exodontia periods. Results are presented as the means of density for bone matrix, osteoblasts and osteoclasts. Also, the results depict the total density of bone tissue was represented by the sum of bone matrix, osteoblasts, and osteoclasts. The results represent the values of the mean and standard deviation in each of the periods analyzed (* represents the differences between the control group).

In this parameter, the bone tissue was represented by bone matrix, osteoblasts, and osteoclasts (**Figure 9A**). In relation to the kinetics, the groups presented the same pattern, with a progressive increase and a higher density in the 14-day period, for all evaluated groups, with no significant differences between them. With respect to the bone matrix, when the 14-day period was compared, a significant increase was observed in the VIP-treated group in relation to the control (**Figure 9B**). Regarding the area density occupied by osteoblasts, there were no significant differences when the control and the PACAP or VIP-treated group were compared (**Figure 9C**). In the group treated with PACAP, an increase was found in the treated groups at 7 days when compared with the control group, and an opposite pattern was observed in the 14-day period when the same groups were compared with a significant decrease in the treated group compared to the control group.

To conclude the bone elements analysis, we observed that the density of osteoclasts was increased in the VIP group in the period of 14 days. In its turn, in relation to the kinetics, it was observed an expected increase only in the period of 14 days verified in all the groups (**Figure 9D**). In the PACAP group, it was observed that the osteoclasts’ density was similar to control group in the 7-day period and significantly lower in the PACAP group when compared to the control group in 14 days (**Figure 9D**). In turn, the increase in osteoclasts’ density observed in the 14-day period for the VIP-treated group showed the beginning of the tissue remodeling process, while the decrease in the PACAP-treated group suggests a faster healing process.

### Collagen Birefringence Analysis

**Figure 10:**
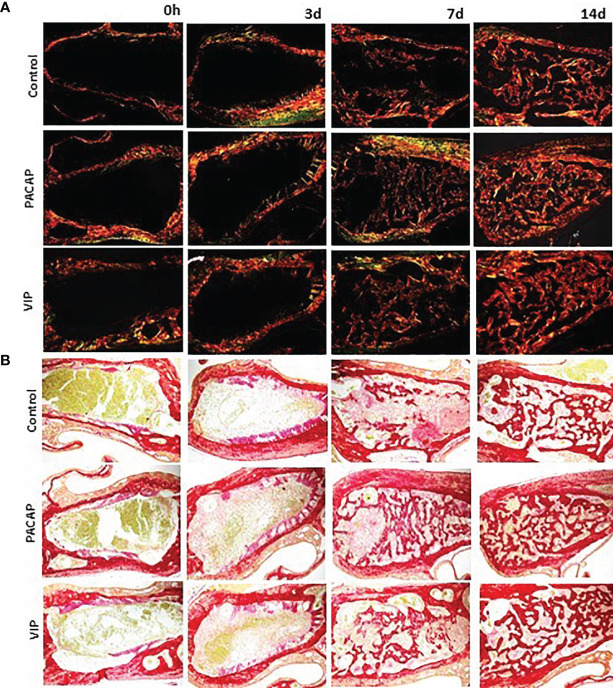
Analysis of birefringence of the collagen fibers present in the dental alveolus in the 0, 3-, 7-, and 14-day post-extraction periods, in the control, PACAP, and VIP groups. Photomicrographs representing the middle region of the dental alveolus, captured under polarized **(A)** and non-polarized light **(B)**, showing the deposition of collagen fibers with birefringence in green, yellow, and red at 0, 3, 7 and 14 days in all the groups (control, PACAP, and VIP). Stain Picrosirius Red; 10× objective.

**Figure 11:**
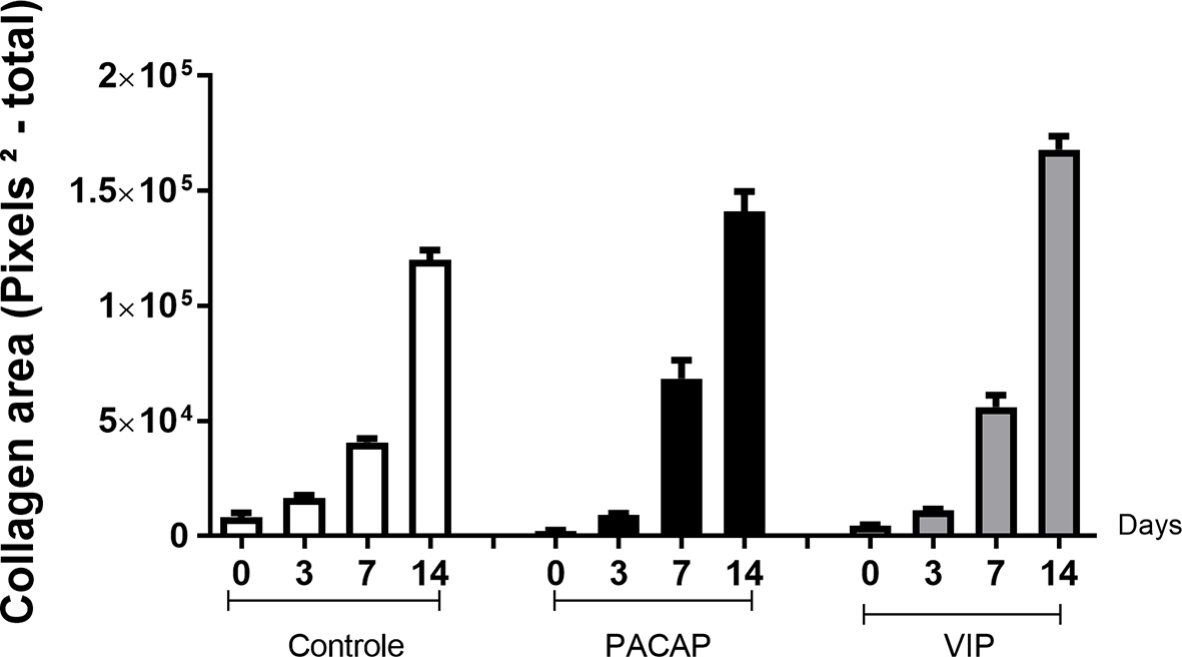
Quantitative analysis of the area (pixels^2^—total) occupied by birefringent collagenous fibers in the dental alveoli at 0, 3-, 7-, and 14-day post-exodontia periods, in the control and treated with PACAP and VIP. Periods of 0, 3, 7 and 14 days represented in relation to the area in pixels^2^ of birefringent fibers relative to the total area, throughout the experimental periods. The results represent the values of the mean and standard deviation in each of the periods analyzed.

**Figure 12:**
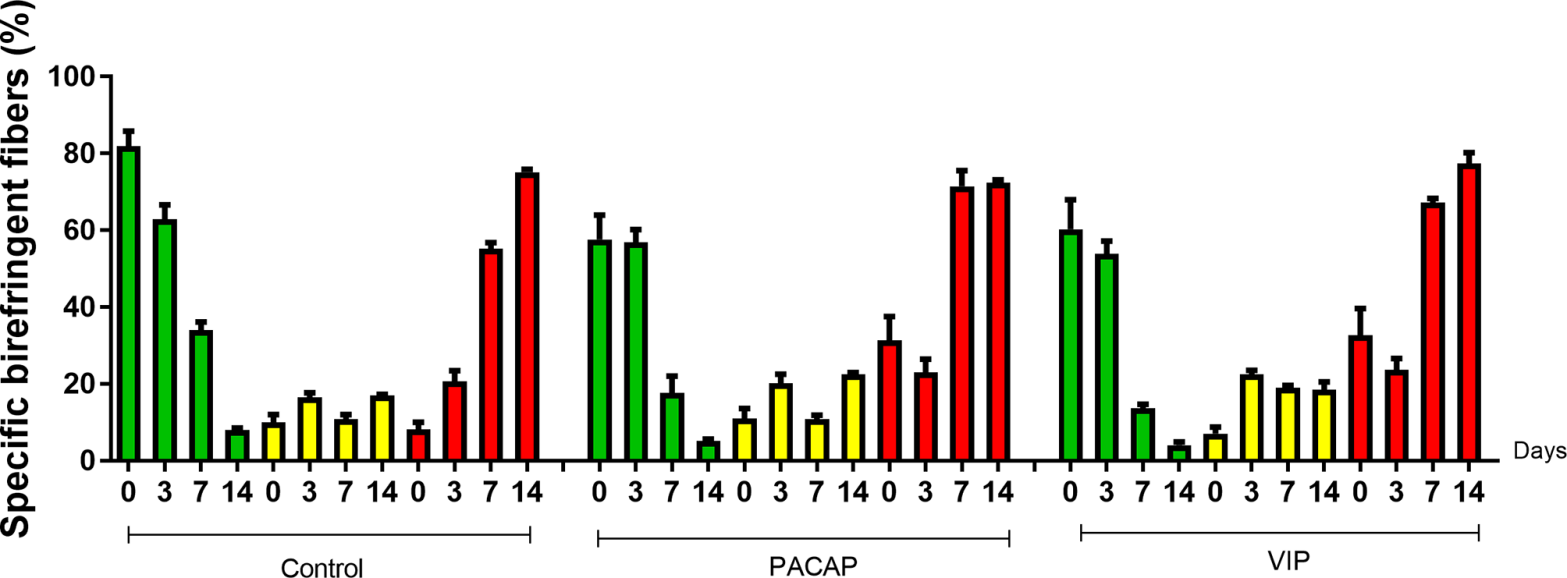
Quantitative analysis of the area (pixels^2^) occupied by green, yellow, and red birefringent collagen fibers in the dental alveolus at 0, 3-, 7-, and 14-day post-exodontia periods, in the control and treated animals with PACAP and VIP. Periods of 0, 3, 7 and 14 days represented in relation to the area in pixels^2^ of birefringent fibers within the green, yellow and red spectrum percentage to the total area, throughout the experimental periods. The results represent the values of the mean and standard deviation in each of the periods analyzed.

Birefringence analysis was used to verify comparatively the formation and maturation of the collagen matrix in the evaluated groups, through collagen fibers stained by Picrosirius Red and which were analyzed under polarized (**Figure 10A**) and non-polarized (**Figure 10B**) light. In the categorization of color spectra, birefringent fibers within the green color spectrum are related to a less organized and immature matrix. In contrast to these, fibers with a color spectrum varying from yellow to red are related to a matrix with a higher degree of organization and maturation (25, 47, 51). Regarding the quantitative analysis of birefringence of the total area (pixels^2^) of the collagen fibers present in the alveoli, it was observed that the number of pixels had a progressive increase in relation to the periods in all groups with no significant differences (**Figure 11**). Regarding the percentage of pixels representing the birefringent fibers in the green, yellow and red spectra, particularly the total quantified pixels in each period, it was observed that the kinetics of the production and maturation of the collagen fibers occurred in a decreasing way for the green fibers and an increasing form for the red fibers. The yellow fibers in turn remain in a smaller quantification that follows during all the periods during the course of the healing process being this pattern observed in all the analyzed groups. In the PACAP and VIP groups, red fibers were increased in the 7-day period compared with the control group, in a quantity similar to that observed in 14 days. However, the yellow fibers in turn remain in a smaller quantification that follows during all the periods during the healing process and showed a little increase in treated groups. Thus, it was observed that the three-color spectra were arranged to form the bone matrix of the newly formed trabeculae in all groups analyzed along with the bone healing, and were without any statistical difference between them (**Figure 12**).

### Analysis of Bone Microarchitecture Parameters by Micro-Computed Tomography (μCT)

**Figure 13:**
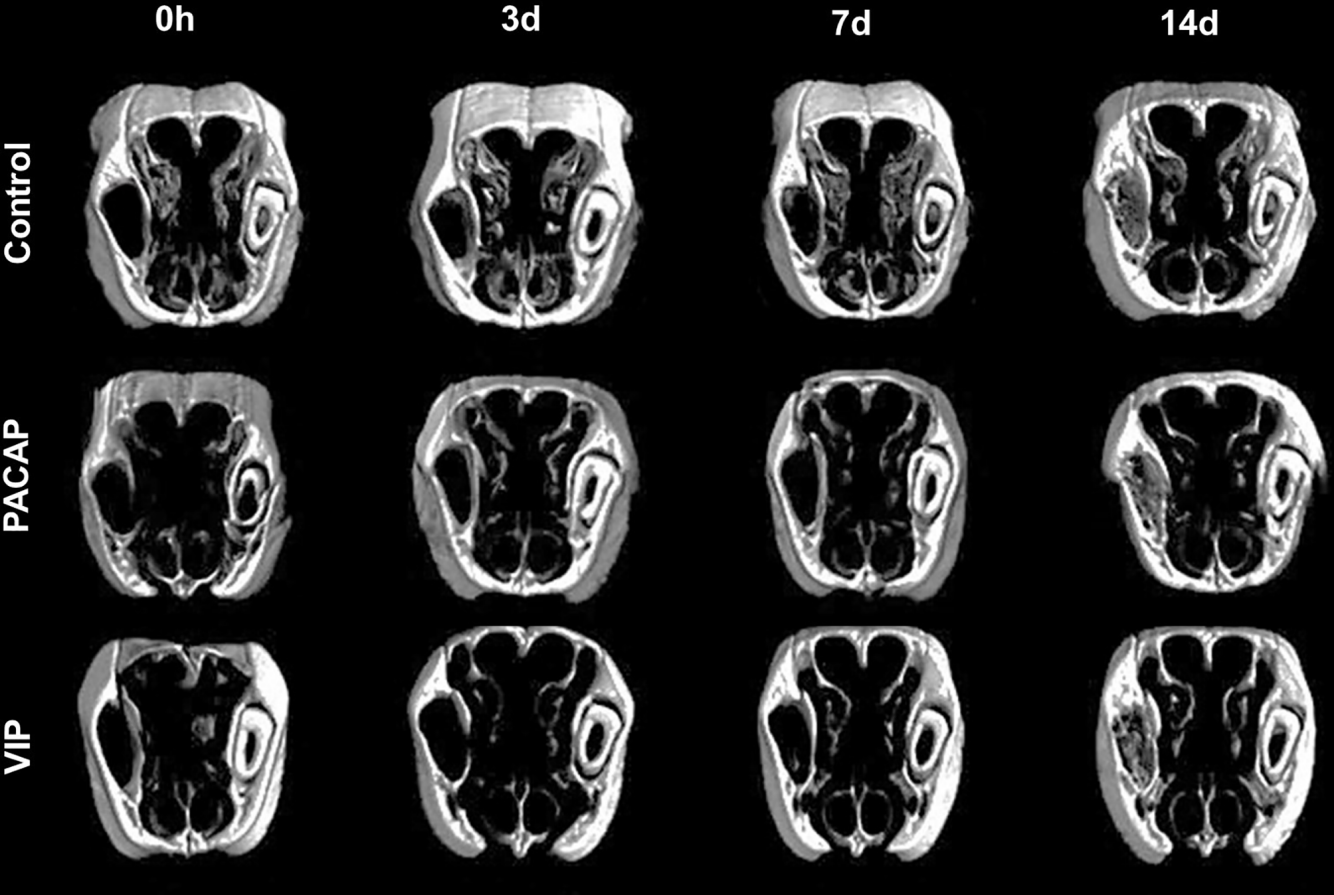
Three-dimensional morphological analysis by µCT of the alveolar bone healing process in mice control and treated with PACAP and VIP in the 0, 3-, 7-, and 14-day post-exodontia periods. The images were reconstructed using the NRecon Reconstruction software and three-dimensional images obtained by the CTvox software. The maxillae were sectioned in the transversal plane.

**Figure 14:**
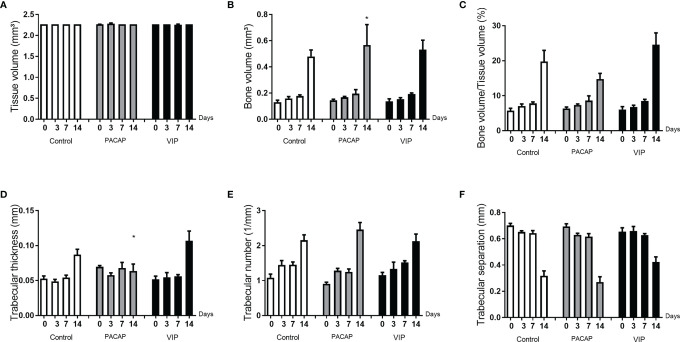
Analysis of the morphological parameters of the trabecular bone microarchitecture in the dental alveoli of the control mice and treated with PACAP and VIP in the 0, 3-, 7-, and 14-day post-exodontia periods. The morphological parameters of the bone microarchitecture were evaluated using the CTAn software through a cylindrical region of interest (ROI) determined by the segmentation of the trabecular bone located from the coronal to the apical region. The analysis of bone parameters **(A–F)** included: Tissue volume (mm^3^) **(A)**, Bone volume (mm^3^) **(B)**, Bone volume in relation to tissue volume (%) **(C)**, Trabeculae thickness (mm) **(D)**, Number of trabeculae (1/mm) **(E)**, and Separation of trabeculae (mm) **(F)**. The results represent the values of the mean and standard deviation in each of the periods analyzed (* represents the differences between the control group).

After the surgeries, maxillae samples from the control and experimental groups were compared based on experimental time (0, 3-, 7-, and 14-day post-exodontia), using three-dimensional reconstruction by CT-Vox software (Bruker, Billerica, Massachusetts, EUA) and our results corroborate with the histomorphometric findings. In all the groups evaluated, it was observed that in the immediate period after extraction the alveolus was completely empty, with an absence of hyperdense areas. Corroborating with previous studies based on the microtomographic analysis (47, 50), we verified that the alveolar bone healing occurred in a centripetal manner, becoming confluent until filling the entire extension of the alveolus (**Figure 13**). Three and seven days after the surgical procedure the presence of discrete regions of bone neoformation close to the cortical bone was observed, following to the central region. At 14 days, a well-evident bone new formation was observed, with a trabeculae formation in the central region, similar between the groups (**Figure 13**). The characteristics of bone microarray were performed employing the CTAn software (Bruker, Billerica, Massachusetts, EUA), and the following parameters were considered: bone volume (BV mm³), tissue volume (TV mm³), percentage of bone in relation to total tissue volume (BV/TV %), trabeculae thickness (Tb.Th mm), number of trabeculae (Tb.N 1/mm), and separation of trabeculae (Tb.Sp mm) (**Figure 14**). In the VIP and PACAP groups, and also in the control group, it was observed that during the periods, there was a progressive increase in bone volume (**Figure 14B**), percentage of bone in relation to total tissue volume (**Figure 14C**), trabeculae thickness (**Figure 14D**), and in the number of trabeculae (**Figure 14E**) and as expected, a decrease occurred in the separation of the trabeculae (**Figure 14F**), following the expected kinetics of the evolution in the process of alveolar bone healing. When the experimental time was compared between the groups, there was no statistically significant variation in the µCT analysis between the VIP-treated and the control group in any of the analyzed parameters. In the PACAP group and also in the control group, it was observed that during the experimental periods, there was an increase in the bone volume, expressive in the 14-day period, with a significant increase between the PACAP and the control group (**Figure 14B**); the progressive increase pattern was also observed with the percentage of bone in relation to total tissue volume for all groups, with no significant differences between them (**Figure 14C**). Regarding trabeculae thickness (**Figure 14D**), significant increases were observed in the PACAP group compared to the control group in 14 days; in the same way, the number of trabeculae was a progressive increase in the PACAP and control groups with no significant differences between them (**Figure 14E**). As expected, there was a decrease in the separation of the trabeculae in 14 days, following the expected kinetics of evolution in the process of alveolar bone healing (**Figure 14F**); when the groups were compared, there was no statistically significant variation between them in the µCT analysis. Importantly, no significant differences were observed in the healing features in males and females, as equivalent numbers were used in the experiments (data not shown).

## Discussion

During the healing process, the host inflammatory immune response contributes to a favorable outcome by coordinating the cellular influx and activity at the injury site (21, 53). Although the exact contribution of leukocyte subsets to the bone healing process is still unclear, studies generally point to macrophages, and their differential acquisition of functional M1 and M2 phenotypes, as key elements of the bone healing process (4, 5). In this context, the present study takes advantage of the immunomodulatory properties of VIP and PACAP, described to favor the development of the M2 phenotype (32, 42–44), to test the impact of M2 polarization in alveolar bone healing, and also investigate the potential mechanisms linking immune response polarization/modulation and bone healing outcome.

Initially, our results demonstrate that control mice data replicates the previous descriptions (13, 15, 17) of an initial M1 dominance, followed by a switch towards a predominant M2-type marker expression, as demonstrated by the gene expression data, and by the shift of putative M1 (CD80^+^) and M2 (CD206^+^) cell dominance over time revealed by immunohistochemistry. While the sole CD80^+^ and CD206^+^ staining do not fully characterize the polarized macrophage phenotypes, the molecular analysis of M1 and M2 markers provides support for polarized response upon VIP and PACAP treatments. In this scenario, we observed that the VIP and PACAP administration were effective in the modulation of M1/M2 balance in the developing healing sites, evidenced by the increased expression of M2-type markers ARG1 and FIZZ along with a decrease in M1 prototypic marker iNOS. Indeed, previous studies demonstrate a M2-polarizing effect of both VIP and PACAP (27, 31, 34, 39, 43, 54). Additionally, a significant increase in CD206^+^ cell counts was observed in both VIP and PACAP groups in the early time-points, usually dominated by M1 cells (i.e., CD80^+^ cells) in the control group (27, 31, 34, 39, 43, 54). Specifically, an early dominance of an M2-type response is observed in both VIP and PACAP groups, and it is sustained in the VIP group at a higher level (when compared to control and PACAP groups) along the healing process. Our data also demonstrate that VIP and PACAP groups presented a decrease in the expression of pro-inflammatory cytokines (IL-1β, IL-6, TNF) and an increase in anti-inflammatory and regulatory mediators (IL-10, TGF-β1), respectively described as characteristic products of M1 and M2 cells (9, 55, 56), supporting the hypothesis of an increased number of M2 macrophages results in an anti-inflammatory environment in the healing sites. Accordingly, VIP and PACAP exert their anti-inflammatory function in several ways by direct inhibition of pro-inflammatory cytokine production such as TNF and IL-6 by activated macrophages; upregulation of IL-10 production; and inhibition of B7.1/B7.2 expression in activated macrophages (57).

Additionally, the impact of VIP and PACAP in the host response is also evidenced by an overall decrease in macrophage (i.e., F4/80^+^ cells) counts in healing sites, even with the increase in the CD206^+^ subset. Such modulation is compatible with the decrease observed in the expression of chemokines (i.e., CCL2, CCL3, and CCL5) and chemokine receptors (i.e., CCR1, CCR2, and CCR5) characteristically involved in the chemotaxis of macrophages (58). Such pattern suggests that VIP and PACAP administration results in the early acquisition of M2 phenotypes by the infiltrating macrophages, which in turn can modulate the subsequent overall response and limit migration of macrophages and M1 phenotype development at healing sites. Notably, while the PACAP group presented a minor decrease in neutrophils/granulocytes (i.e., GR1^+^ cells), VIP administration resulted in increased GR1^+^ cells cell counts. Accordingly, a significant increase in the expression of neutrophil-related chemokine receptors (CXCR1, CXCR3) was observed in the VIP group, as well in the MMP8 (considered a neutrophil activity marker), suggesting an increase in neutrophil activity in the healing sites. While the exact effects of VIP on neutrophils remain unclear, studies suggest a PACAP can present an inhibitory effect on chemotaxis (59–61). While the role of neutrophils in bone regeneration remains elusive, the deficiency or excessive migration and permanence of neutrophils in the tissues have a negative impact on the repair process (62–64). In addition, recent studies suggest that neutrophils can play pivotal roles in orchestrating host response in the initial stage of bone healing (65).

Noteworthy, CD3^+^ counts remain low in the VIP and PACAP groups resembling the control group, despite the increased expression of chemokines related to lymphocyte migration (i.e., CCL12, CCL17, and CCL20). It is important to consider that lymphocytes are usually present at low counts in bone healing sites; however, they are extremely important during the repair process, acting directly on the balance between RANKL and OPG (66–73). Thus, the presence of VIP and PACAP in the healing site is able to modulate the activity of T lymphocytes through functions that include vasodilation and cell migration, with the capacity to regulate pro- and anti-inflammatory mediators (74). At this point, is possible to consider that the anti-inflammatory effects of VIP and PACAP may counteract the increase in chemokine levels, limiting the cell infiltration in the healing sites. In this context, it is also worth mentioning that MSCs related chemokine CXCL12, and MSCs markers (i.e., CD106, CD166, OCT-4, CD34, and CD146 were also increased by VIP and PACAP, suggesting a potential for improved healing in this groups, comprised by both signals for osteogenic differentiation and cells prone to undergo this process (25).

Therefore, our initial analysis confirm the immunoregulatory effect of both VIP and PACAP at healing sites, which modulated a series of host response related markers and resulted in an M2-polarizing effect (27, 31, 34, 39, 43, 54), allowing the subsequent analysis of the M2 polarization impact in alveolar bone healing outcome. However, despite the marked effect of M1/M2 balance in the healing sites, the histomorphometric analysis does not reveal an equivalent/corresponding modulation of the healing process, contradicting the original hypothesis that an early and/or increased M2 response could improve healing outcome to a large extent.

For successful healing, the first stage consists of the formation and subsequent maturation of the blood clot (25). Even with the VIP and PACAP administration prior to the tooth extraction and previous descriptions of possible PACAP influence in platelet activation (75, 76), no effects were observed in the initial clot formation, followed by a normal transitional formation of a connective tissue that will support the subsequent healing stages (25, 77, 78). In the early healing stages, a decrease in blood vessels in the VIP group was revealed by the histomorphometric analysis, which parallels a decrease of VEGFA expression in the same group. Indeed, in homeostatic conditions, previous studies demonstrate that VIP can increase angiogenesis by the upregulation of VEGF (79, 80), which cannot be observed through our results.

As regards subsequent connective tissue formation, the histomorphometric analysis reveals slight changes in the density of fibroblasts and collagen fibers density, while the birefringence analysis suggests an increase in collagen fibers density in the VIP and PACAP-treated groups in the period of 7 days. However, the molecular analysis did not show evidence of significant variations in the collagen mRNA levels upon treated and control groups, despite the previous description that VIP can increase fibroblasts proliferation and collagen synthesis in the skin (81). Noteworthy, a significant decrease in MMPs (i.e., MMP1a, MMP2), involved in ECM matrix remodeling was observed in VIP and PACAP groups, which can be derived from its anti-inflammatory effect. With well-established functions, MMPs are part of a group of proteins secreted as proenzymes released by neutrophils, monocytes, macrophages, and fibroblasts, being of fundamental importance in tissue remodeling and healing, in addition to the structural and functional maintenance of tissues (82, 83). Indeed, M1-associated pro-inflammatory cytokines, found to be decreased in VIP and PACAP groups, are classically described to increase MMPs levels (69). Importantly, while VIP and PACAP treatments seem to reduce MMP levels in healing sites, their expression is still detectable, and probably accounts for the ECM remodeling that takes place during the healing process.

Considering the bone formation as the main healing readout, we next evaluated bone parameters/readouts by µCT and histomorphometric analysis. µCT analysis reveals a slight (but statistically significant) increase in the bone matrix volume and the trabecular thickness number was observed at the 14-d time-point in the PACAP group, while histomorphometric analyses reveal that the bone matrix density was slightly increased in the VIP group at the 14-d time-point.

At this point, it is also mandatory to consider that the sensibility of molecular analysis, such as the RealTimePCR performed, may be higher than the µCT and histomorphometric methods, which may not reflect alterations in bone healing that took place to a lower degree. Indeed, the molecular analysis demonstrates that both VIP and PACAP administration results in an increased expression of osteogenic factors (i.e., BMP2, BMP4, and BMP7), and also of osteoblastic differentiation prototypic transcription factors (i.e., RUNX2) and activity markers (i.e., ALP). Another molecular evidence of increased bone maturation in the VIP and PACAP groups derives from the increased expression of osteocyte markers DMP1, Phex, and SOST. Accordingly, previous bodies of evidence support the anabolic effect of VIP and PACAP over bone cells/tissue (24, 84), which includes the activation of transcription factors such as Runx2 (85), and the production of ALP, osteocalcin, and osteopontin, essential to proper bone neoformation and maturation (86). Also, the decrease of osteoclast density provided by the treatment with PACAP, corroborating with previous studies that showed specific receptors to VIP and PACAP in these cells (87, 88), and the increased expression of OPG and the decrease in CTSK, which are characteristic markers of bone remodeling and osteoclastic activity (89, 90), in the treated group reinforce these bodies of evidence.

Importantly, among a range of neuropeptides and neurotrophins, VIP and PACAP are listed as elements involved in bone regeneration and callus formation, and their absence during repair makes it significantly impaired with decreased bone callus density and mechanical strength (91). The absence of PACAP promotes a decrease in the expression of collagen type I and BMPs, hindering the formation of callus (92), suggesting that it favors osteoarthritis (93) and other joint diseases (94). Complementary, VIP is shown to be able to modulate the interactions between nerve fibers and bone cells, through the expression by osteogenic cells of factors responsible for the maintenance of the periosteum (95, 96). However, unique features of endochondral and intramembranous healing may account for distinct roles of VIP and PACAP in these processes. Indeed, the absence of increased Col2a1 expression, a cartilage specific ECM component and characteristically upregulated in endochondral healing (97), reinforce the intramembranous nature of alveolar bone repair.

Importantly, VIP and PACAP are naturally expressed during alveolar bone repair, in accordance with previous studies that demonstrate the neuropeptides in bone cells interplay (41, 88, 98), and these factors could account/contribute to the natural M2 phenotype acquisition during bone healing. Interestingly, we observed a lower number of VIP and PACAP immunolabeled in the treated groups, which suggests a reduction in endogenous production during treatments. Accordingly, a complex regulatory interplay seems to take place between VIP, PACAP, and its receptors (99, 100). Therefore, even with the administration of exogenous recombinant VIP or PACAP, the total local levels may not be significantly increased in view of the endogenous production decrease. However, it is necessary to consider that despite the uncertainty regarding endogenous and exogenous VIP/PACAP putative cross-regulation, the M2 response readouts are increased in the VIP and PACAP groups.

Therefore, since both µCT and histomorphometric analysis do not provide consistent evidence for a significant modulation of bone healing, it is possible to conclude that VIP and PACAP treatments only resulted in minor changes in the bone healing outcome. In this context, we hypothesize that despite a potential for increased healing, the signals required for bone healing under homeostatic conditions are already ‘optimal’, and additional signals do not improve an already ‘optimal’ process. In this framework, we must consider that the inflammatory cell migration that takes place in control mice is considered ‘controlled’, and is naturally subjected to some degree of immunoregulatory control of M2 that are present in the inflammatory infiltrate, where in its natural course, the healing process is already regulated, undergoing modulations in the amount of M1 and M2 cells according to the healing stage (13). In this sense, the modulation observed by the treatments with VIP and PACAP did not result in additional benefits to healing itself. Indeed, when recombinant VIP is administered in non-homeostatic/pathological conditions (i.e., in a model of infectious inflammatory bone lesions), it results in a clear modulation of host response, which includes M2-switch but is translated in a clear phenotypic change in the lesions (13).

Noteworthy, the VIP modulating capacity for an M2 profile was observed more clearly in acute inflammatory processes of bone tissue (49, 101, 102) and cells previously stimulated for an M1 profile (103–105), or even in the dental context in the previous studies of our group where the treatment with VIP in periapical lesions was analyzed (106). Therefore, the lack of a direct correlation between the increased M2 activity and bone healing does not rule out the pro-healing role of M2 macrophages, and it is possible that while VIP/PACAP immunomodulatory properties can reserve a pathological pro-inflammatory environment towards a pro-reparative milieu, its administration in homeostatic/normal bone healing conditions, which are naturally a pro-reparative milieu, does not significantly modify the healing phenotype/outcome or that under homeostatic/normal conditions, the endogenous production of VIP and PACAP will account for optimal immunoregulatory activity.

In conclusion, the present study demonstrated an increase in the bone tissue in the VIP and PACAP treated group at the endpoint. Our results demonstrate that the early healing observed in the VIP and PACAP groups was associated with the increased presence of M2 macrophages and associated labels. Further studies are required to clarify the underlying mechanisms and influence of VIP/PACAP during bone healing.

## Data Availability Statement

The original contributions presented in the study are included in the article/supplementary material. Further inquiries can be directed to the corresponding author.

## Ethics Statement

The animal study was reviewed and approved by the Institutional Committee for Care and Animal Use and by Guide for the Care and Use of Laboratory Animals (CEEPA-FOB/USP #01/2016).

## Author Contributions

MA and GG contributed to the conception and design, the acquisition, analysis, and interpretation, drafted the manuscript, critically revised the manuscript, gave final approval, and agreed to be accountable for all aspects of the work. AF, PC, JM, APT, and APFT contributed to the acquisition, analysis, and interpretation, gave final approval, and agreed to be accountable for all aspects of work. AC and AM contributed to the acquisition, analysis, and interpretation, critically revised the manuscript, gave final approval, and agreed to be accountable for all aspects of work. All authors contributed to the article and approved the submitted version.

## Funding

This study was supported by scholarship grants #2015/25618-2 and #2015/24637-3 from São Paulo Research Foundation (FAPESP), CNPq, and CAPES.

## Conflict of Interest

The authors declare that the research was conducted in the absence of any commercial or financial relationships that could be construed as a potential conflict of interest.

## Publisher’s Note

All claims expressed in this article are solely those of the authors and do not necessarily represent those of their affiliated organizations, or those of the publisher, the editors and the reviewers. Any product that may be evaluated in this article, or claim that may be made by its manufacturer, is not guaranteed or endorsed by the publisher.
